# Hospital Admission Profile Related to Inner Ear Diseases in England and Wales

**DOI:** 10.3390/healthcare11101457

**Published:** 2023-05-17

**Authors:** Esra’ O. Taybeh, Abdallah Y. Naser

**Affiliations:** Department of Applied Pharmaceutical Sciences and Clinical Pharmacy, Faculty of Pharmacy, Isra University, Amman 11622, Jordan

**Keywords:** admissions, ear, England, hospitalisation, inner ear, Wales

## Abstract

Background: Due to an expansion in the usage of medications (such as anticancer therapies), increased exposure to noise, and an increase in life expectancy, the prevalence of inner ear disease-related hearing loss is rising. Diseases of the inner ear are frequently accompanied by other conditions, such as chronic heart failure, systemic inflammation, arterial hypertension, and cerebrovascular disease. The aim of this study was to investigate the profile of hospital admissions linked to inner ear diseases in England and Wales. Method: This was an ecological descriptive study using public medical databases in England and Wales. Diagnostic codes for diseases of the inner ear (H80–H83) were used to identify all hospital admissions. Between 1999 and 2020, the chi-squared test was used to assess the difference between the admission rates. Results: From 5704 in 1999 to 19,097 in 2020, the total annual number of hospital admissions increased by 234.8%, which corresponds to a 192.3% increase in the admission rate [from 10.94 (95% CI 10.66–11.22) in 1999 to 31.98 (95% CI 31.52–32.43) in 2020 per 100,000 people, *p* < 0.01]. “Disorders of vestibular function” and “other inner ear diseases” were the most frequent causes of hospital admissions due to inner ear diseases, accounting for 47.6% and 43.6%, respectively. The age range of 15 to 59 years accounted for 42.3% of all diseases of the inner ear hospital admissions. Around 59.6% of all admissions were made by females. The female admission rate increased by 210.1% (from 12.43 (95% CI 12.01–12.85) in 1999 to 38.54 (95% CI 37.84–39.24) in 2020 per 100,000 people). The male admission rate for diseases of the inner ear increased by 169.6% [from 9.37 (95% CI 9.00–9.75) in 1999 to 25.26 (95% CI 24.69–25.84) per 100,000 people] in 2020. Conclusion: Inner ear disease admissions increased markedly in England and Wales during the past two decades. Females and the middle-aged population were at higher risk of being admitted for inner ear diseases. Further cohort studies are warranted to identify other risk factors and develop effective prevention strategies.

## 1. Introduction

Ear diseases are varied and can influence the sense of balance and hearing. Ear disorders can be categorised according to the part of the ear affected, i.e., the inner, middle, or external ear [1,2]. The innermost part of the ear is the inner ear, which includes the organs responsible for the sense of hearing and balance. The inner ear is located in the temporal bone and comprises three primary parts: the semicircular canals, the vestibule, and the cochlea [1,2].

Inner ear disorders involve the entire membranous labyrinth and are distinguished by the following three symptoms: vertigo, tinnitus, and sensorineural hearing loss. Primary pathology may include aberrant inner ear homeostasis, supporting cells, or inner ear hair cells leading to an alteration in the composition of the peri and endolymph, with direct influences on the function and integrity of the hair cells [3]. Changed efferent and afferent auditory pathways may be the main reason for inner ear symptoms or may escort a diseased inner ear [3].

The most common inner ear-related dysfunction is age-related hearing loss [2]. Hearing loss affects more than 1.5 billion people worldwide [4]. By 2025, the number of people with hearing loss could rise to more than 2.5 billion [4]. Inner ear disease-related hearing loss prevalence is increasing due to an increase in the use of medicines (like anticancer drugs), noise exposure, and life expectancy [5]. Hearing impairment can significantly impact many aspects of an individual’s life, including employment and educational opportunities, physical and mental wellbeing, and socioeconomic status [6]. Worldwide, unaddressed hearing loss has a total annual cost of 980 billion dollars [4].

The existence of diseases of the inner ear is usually associated with other diseases and health problems, including chronic heart failure, systemic inflammation, arterial hypertension, cerebrovascular disease, diabetes, chronic kidney disease, asymptomatic atherosclerosis, obesity, coronary artery disease, and dyslipidaemia [7]. In addition, one of the main causes of ear pathology, even inner ear pathology, is infectious disease [8]. Moreover, the treatment results of diseases of the inner ear remain poor as they are intractable [5]. There have been no previous studies that explored hospital admission patterns related to inner ear diseases. Previous studies examined other common diseases in the UK [9,10,11,12]. We aimed to study hospital admission profiles related to inner ear diseases in England and Wales.

## 2. Methods

### 2.1. Data Source and the Population

This was an ecological descriptive study that was conducted using publicly available data for the period between April 1999 and April 2020 extracted from two medical databases in the United Kingdom: the “Hospital Episode Statistics” (HES) database in England and the “Patient Episode Database for Wales” (PEDW) [13,14]. We did not include the data for 2021 due to the COVID-19 pandemic, which has impacted the admission trends for different diseases. Data in these two databases are reported stratified by gender and by age. Age groups are subdivided into four categories: “below 15 years, 15–59 years, 60–74 years, and 75 years and above”. We identified diseases of inner ear-related hospital admissions using the “Tenth Revision of the International Statistical Classification of Diseases and Related Health Problems” (ICD-10) 5th Edition (H80–H83) [15]. The hospital admission rate was calculated using mid-year population data, which was extracted from the Office for National Statistics (ONS).

### 2.2. Statistical Analysis

Admission rates were estimated using the number of finished consultant episodes of diseases of the inner ear-related admission divided by the mid-year population and presented with 95% confidence intervals (CIs). The age-specific admission rate was estimated by dividing the number of admission episodes for each age group by the mid-year population of that same age group in the same year. The same procedure was followed to estimate the gender-specific admission rate by dividing the number of admission episodes for each gender (males or females) by the mid-year population of males or females in the same year. The chi-squared test was employed to estimate the difference between the admission rates between 1999 and 2020. Analyses were conducted using SPSS version 27 (IBM Corp, Armonk, NY, USA).

## 3. Results

The total annual number of diseases of the inner ear hospital admissions for various causes rose by 234.8%, which started from 5704 in 1999 and reached 19,097 in 2020, representing an increase in the admission rate of 192.3% [from 10.94 (95% CI 10.66–11.22) in 1999 to 31.98 (95% CI 31.52–32.43) in 2020 per 100,000 persons, *p* < 0.01].

The most common causes of admission were “disorders of vestibular function” and “other diseases of inner ear”, which accounted for 47.6% and 43.6%, respectively (Table 1).

**Figure 1:**
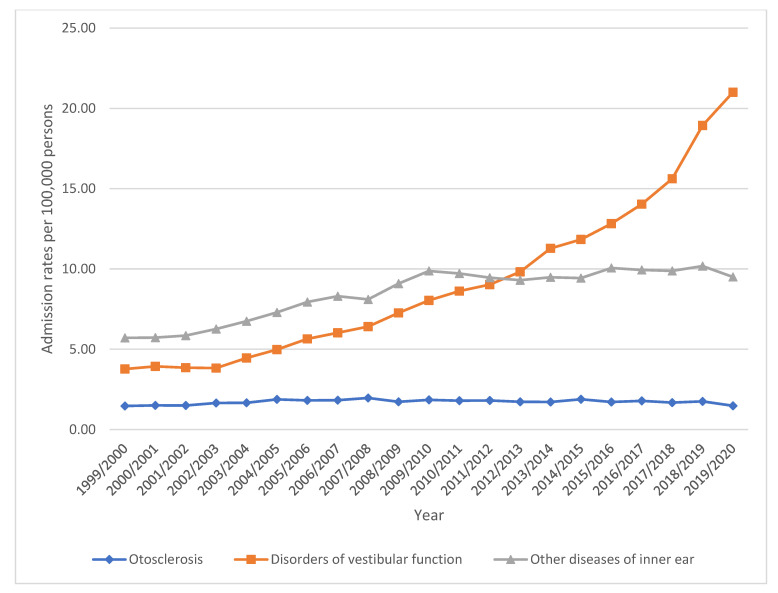
Admission rates stratified by type between 1999 and 2020.

Over the study duration, a tremendous increase in disease of the inner ear hospital admission rates was seen in disorders of vestibular function by 457.5%. Besides, hospital admission rates for other diseases of the inner ear and otosclerosis were increased by 66.5% and 0.7%, respectively (Figure 1 and Table 2).

The most common causes of admissions were “labyrinthitis”, “benign paroxysmal vertigo”, and “Ménière disease”, which contributed 41.4%, 23.2%, and 12.0%, respectively. The highest increase in the admission rate for inner ear diseases was for “benign paroxysmal vertigo” (2160.3%). This was followed by “other peripheral vertigo”, “other disorders of vestibular function”, and “vestibular neuronitis”. For further details, refer to Table 3.

**Figure 2:**
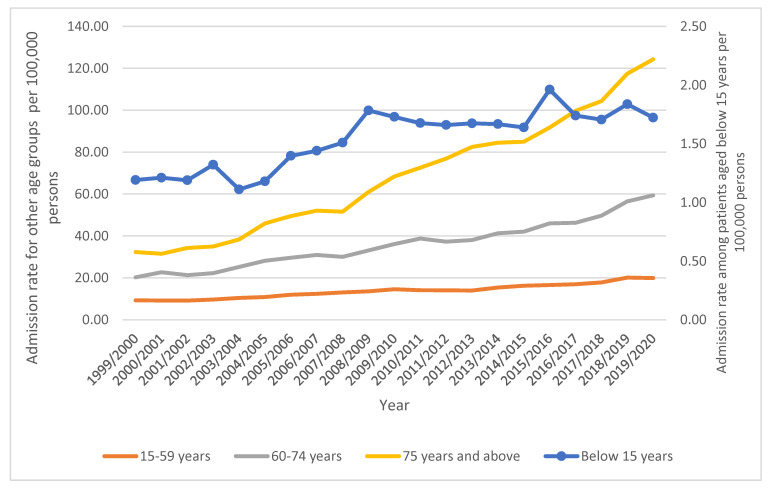
Admission rates stratified by age.

Most hospital admissions were for the age group 15–59 years (42.3%) and the age group 75 years and above (28.8%), Table 4. The lowest increase in the rate of hospital admission was observed among patients aged below 15 years (44.6%). The highest increase in the rate of hospital admission was observed among patients aged 75 years and above (284.9%) [from 32.29 (95%CI 30.51–34.07) in 1999 to 124.28 (95%CI 121.24–127.31) in 2020 per 100,000 persons]; Table 3. Figure 2 presents admission rates stratified by age group.

**Figure 3:**
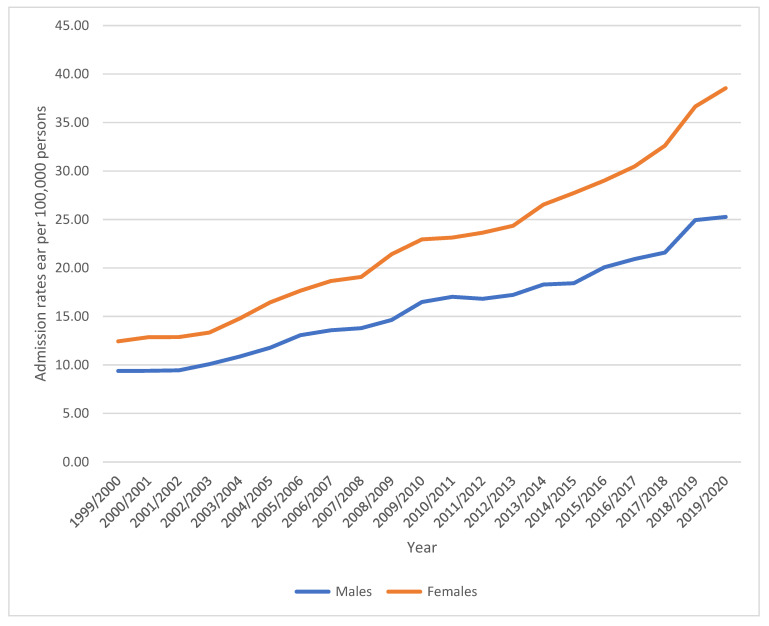
Admission rates stratified by gender.

A total of 228,733 admission episodes were reported during the study timeframe. Females contributed to 59.6% of the total number of admissions (136,224 admission episodes), with an annual average of 6486 episodes. The female admission rate rose by 210.1% [from 12.43 (95% CI 12.01–12.85) in 1999 to 38.54 (95% CI 37.84–39.24) in 2020 per 100,000 persons]. The male admission rate increased by 169.6% [from 9.37 (95% CI 9.00–9.75) in 1999 to 25.26 (95% CI 24.69–25.84) in 2020 per 100,000 persons] (Figure 3).

### 3.1. Diseases of the Inner Ear Admission Rate by Gender

**Figure 4:**
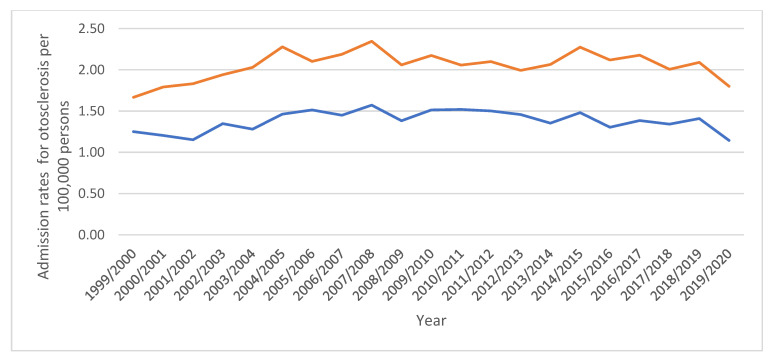

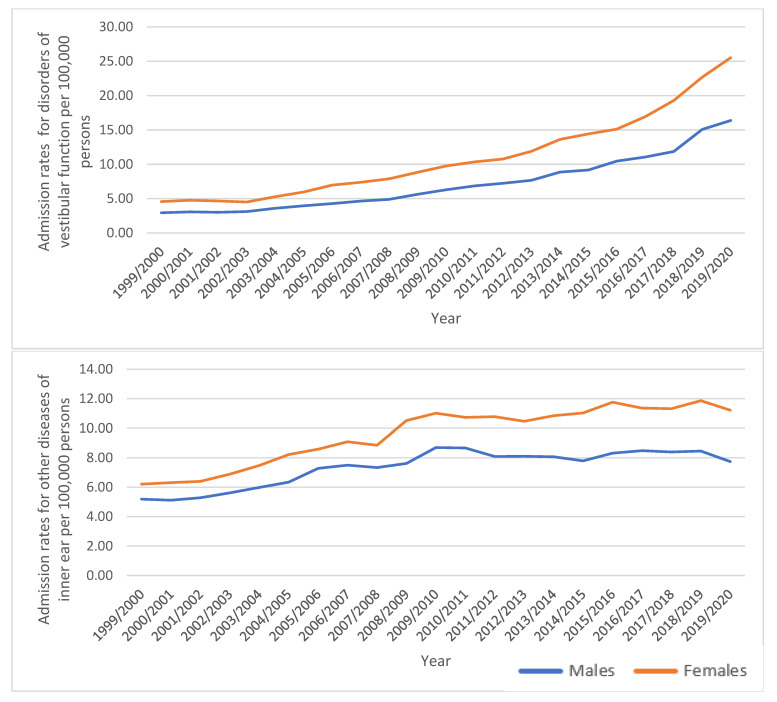
Admission rates stratified by gender and indication.

Admission rates for all inner ear diseases were higher among females compared to males (Figure 4).

### 3.2. Diseases of the Inner Ear Admission Rate by Age

**Figure 5:**
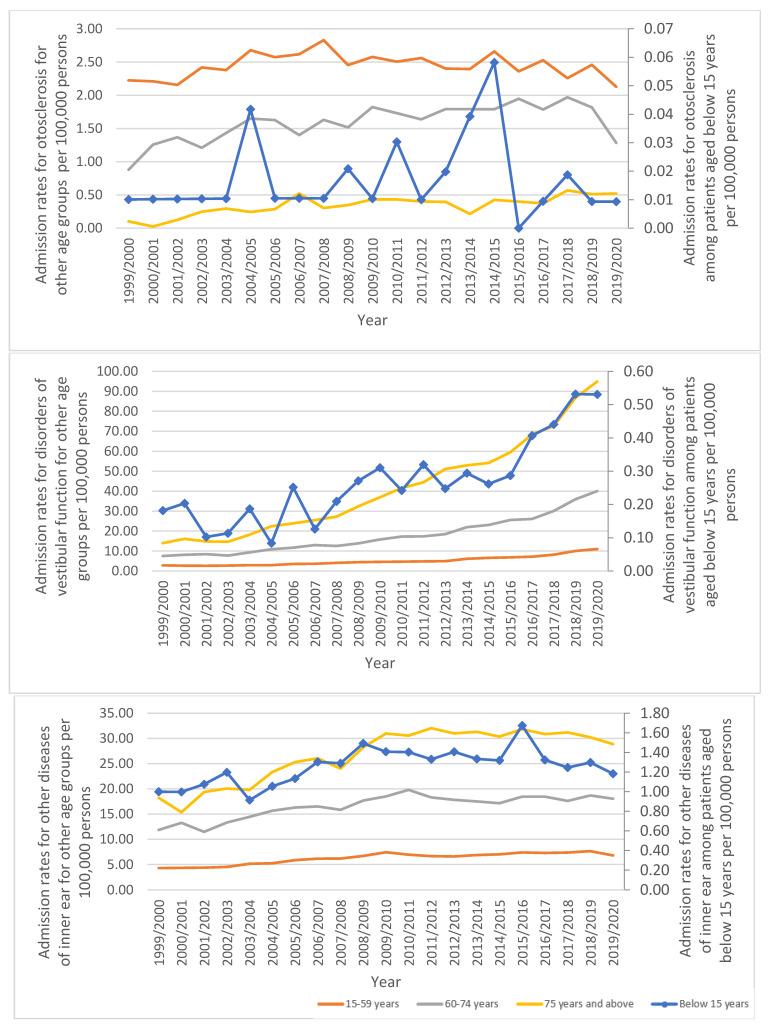
Admission rates stratified by age and indication.

Diseases of the inner ear hospital admission rates for disorders of vestibular function and other diseases of the inner ear were directly related to age (more common among elderly patients aged 75 years and above) (Figure 5).

## 4. Discussion

This research is the first nationwide epidemiological study in the United Kingdom (England and Wales) to investigate the changes in hospital admissions and admission rates for inner ear disorders with comparison by age and gender over the period 1999–2020 based on representative data from the HES and PEDW medical databases.

Our finding showed that the total annual number of diseases of the inner ear hospital admissions for various causes increased by 234.8% from 5704 in 1999 to 19,097 in 2020, representing an increase in hospital admission rate of 192.3% [from 10.94 (95% CI 10.66–11.22) in 1999 to 31.98 (95% CI 31.52–32.43) in 2020 per 100,000 persons, *p* < 0.01]. The increased admission rates can be justified by the increased prevalence rates of diseases of the inner ear with time. For instance, the 2003 prevalence of vestibular vertigo in Germany, as reported by Neuhauser et al. (2009), was 4.9%, while it was 6.5% in 2015 [16,17]. Similarly, Lai et al. (2011) reported a prevalence of 3.1% of vestibular vertigo in Taiwan, while a higher documented estimated prevalence rate (5.0%) was reported three years later [18,19]. Indeed, laboratory testing for inner ear disorders has improved in recent years, and consequently, vestibular apparatus examination has become feasible, and vestibular disorders, therefore, can be diagnosed more frequently [20].

One of the major otologic complaints among Emergency Department (ED) visitors is dizziness and vertigo [21]. While the majority of the patients who were evaluated in the ED for otologic complaints (98.2%) are discharged home [22], only those with severe symptoms are admitted. Yet, the severity of otologic related symptoms explains why disorders of the inner ear are the most common among ear disease hospital admissions. Moreover, common inner ear causes of otologic symptoms are related to vestibular dysfunction [23]. This later disorder was the main cause of hospital admission in our study, and it comes in line with the study of Renner et al. (2017), where “Ménière’s disease, benign paroxysmal vertigo, vestibular neuronitis, vertigo of central origin” were the dominant diagnoses among inpatient patients [24]. Previous studies have consistently shown that the most common causes of these disorders are benign paroxysmal positional vertigo, vestibular neuronitis, and Meniere’s disease [25,26,27]. This was confirming our study findings that the most common causes of admissions were “labyrinthitis”, “benign paroxysmal vertigo”, and “Ménière disease”, which contributed 41.4%, 23.2%, and 12.0%, respectively.

Another important cause of inner ear disease complications is the presence of infectious diseases [8]. Multiple ear-related complications, such as hearing loss, have been reported in the literature to be associated with various types of bacterial and viral infections [8]. A previous study in the UK reported an increase in hospital admissions due to various types of infectious diseases [28].

While middle ear diseases are clearly diseases of children [29], the inpatient inner ear disorders were disorders of older age (42.3% among 15–59 years, and 28.8% among older than 75 years of age). Hospital admissions due to otosclerosis were more common among 15–59-year-old patients; this can be confirmed by a previous study, which found most participants (62%) reported the age of onset of the otosclerosis disease in the third and fourth decades [30]. Patients with otosclerosis, which is a condition that causes conductive hearing loss because of abnormal bone growth in the middle ear, might be admitted to hospitals for surgical intervention, diagnostic testing, monitoring of the disease, and rehabilitation after treatment [31]. On the other hand, hospital admission rates for disorders of vestibular function and other diseases of the inner ear in our study were more common among the elderly. Consistent with the study of Ekwall et al. (2016), which reported that inner ear-related dizziness is one of the most frequent chief complaints among elderly patients presenting in the ED and that such patients often call for an ambulance [32]. The observed increments in hospital admissions at older ages, illustrated in the present study, can be explained by age-related declines in the function of the vestibular system and its consequences of imbalance and falls. Hülse and colleagues (2019) [16], for instance, reported that the prevalence of vestibular disorders increased with age and peaked between 74 and 94 years. Previous literature has identified that vestibular disorders increase with age [33,34,35]. Moreover, it is possible that gout, a condition that is more commonly observed in elderly individuals, is associated with vestibular disorders by mechanisms such as purine crystal deposits accumulation from free-floating otoconial debris in the semicircular canals, gelatinous matrix inflammation linked to otoconia, the production of harmful reactive oxygen species, and the release of inflammatory mediators resulting from elevated serum uric acid levels [36].

Our findings showed that all diseases of the inner ear hospital admissions were higher among females and thus confirm that females are more susceptible to inner ear disorders, which has already been revealed in previous epidemiological studies [16,35,37]. This can be attributed to multiple possible explanations: anxiety-evoked dizziness, where the mechanism is explained by a response to stressful environmental challenges through chronic vasopressin administration to guinea pigs, which leads to endolymphatic hydrops in a mechanism mimicking Meniere’s disease pathology [38]. Interestingly, the prevalence of anxiety disorders was reported to be higher for females (23.4%) than for males (14.3%), which can justify why women are more likely to suffer from vestibular dysfunction [39]. Not only anxiety but also a higher incidence of osteoporosis and demyelinating diseases among women are associated with developing inner ear disorders and warrant further investigation [40,41]. Evidence suggests a correlation between bone mass at peripheral and central sites and that bone demineralization in the otic capsule and cochlea in patients with osteoporosis may lead to neuronal degeneration and sensorineural hearing loss [42,43]. In the UK, it is estimated that 6.7% of males and 21.9% of females who are aged over 50 have osteoporosis [44]. A previous study by Kahveci and colleagues reported that patients diagnosed with osteoporosis have a higher incidence rate of hearing loss (sensorineural type) and that Cochlear dysfunction might play a role in this process [45].

Hormonal influences might also provide a partial explanation for our findings. Research on benign paroxysmal positional vertigo suggests that the rapid decrease in estrogen level disrupts otoconial metabolism within the inner ear and that hormonal therapeutic intervention successfully improves Ménière’s disease [46]. Typical complaints in emergency departments are vertigo, unsteadiness, and imbalance. It is essential to distinguish between central and peripheral etiologies [47]. It is difficult to distinguish between benign vestibular disorders and dangerous brain diseases. Typically, physicians resolve this diagnostic challenge based on cardio-vascular comorbidities. This strategy has raised concerns due to the potential for excessive neuroimaging. Initial diffusion-weighted MRI of the brain is the gold standard [47].

Hearing impairment (HI) is becoming more and more of a public health issue. Nearly two-thirds of the elderly population in the US has hearing impairment [48,49], yet less than 20% of people with hearing loss get any kind of therapy [50]. A variety of detrimental health consequences, including falls, cognitive decline, depression, dementia, and an increase in hospitalizations, have all been associated with HI, which is significant. Elderly adults with hearing loss annually incur additional medical costs of $3.3 billion [51]. This sum is an excess of $420 in medical costs per person per year. A study from The Netherlands estimated that primary, secondary, and occupational health care related to HI together accounted for an excess of $700 in medical spending per person. According to other research, the financial burden of hearing loss is much higher. Another study of older people estimated that hearing therapy costs $12.8 billion nationwide during the first year of therapy [52] in medical expenditures, including audiometric testing and treatment with bilateral hearing aids. In another study, the lifetime medical expenses for HI were stratified by age [53]. Lifetime medical costs associated with hearing loss were $33,794 per person for hearing loss that began at age 65 or older vs. $79,343 per person for hearing loss that began at a younger age (18–44 years). Australia’s annual medical costs for HI were calculated to be $706 million [54].

Indicating the effect of a disease or disability by counting the number of years of healthy life lost owing to illness or disability, the disability burden was most often stated in DALYs. According to Australian research, there are 48 million DALYs overall and 95,000 DALYs from HI per year [54,55]. A total of 559,200 YLDs of lifetime illness burden are included in US estimations. According to estimates, the worldwide burden of hearing loss is 1.3% of the total global illness burden, or 7.5 million DALYs.

The financial burden of filing disability claims with the government or receiving workers’ compensation was a major factor in a lot of the studies on the economic effects of noise-induced hearing loss. Hearing loss accounted for 5.3% of all disability claims in the US, placing it second only to tinnitus [56]. The amount of money paid out for all audiologic complaints is estimated to reach $4.38 billion, of which $1.8 billion was for hearing loss. In Washington State, an examination of 6539 nonfederal workers’ compensation applications over 8 years revealed total disability benefits of $45.6 million [57]. Another study estimated projected lifetime expenses of $16,289 per patient and anticipated degrees of hearing damage with various levels of noise exposure that would be experienced in the US Navy [58].

In addition to the expense of hearing loss treatment, such as audiologic exams and hearing aids, the causes of excessive medical spending among people with HI may be connected to an increase in falls, hospitalizations, cognitive decline [59], and depression [60].

In the United States alone, excess medical expenses for people with hearing loss are expected to range from $3.3 to $12.8 billion [51,52]. Foley et al. [51] and Stucky et al. [52] determined the cost of the first year of hearing health care treatment for elderly adults with HI, and then this cost was extrapolated nationally using a model that took into account self-reported hearing loss. While Stucky et al. estimate the cost of medical therapy assuming all individuals with HI sought treatment [52], the research by Foley et al. [51] likely reflects a more accurate evaluation of the genuine extra cost of HI to the medical system.

Estimating indirect expenses is more challenging. Differences in employment rates and income inequalities may be used to estimate lost productivity. From $1.8 to $194 billion [52,61], various estimates of the economic cost of lost productivity have been made.

The database used to conduct the present work is a useful data source to provide epidemiological information on hospital admissions for inner ear disorders. There could be a potential source of miscoding regarding the diagnoses by health care providers, although miscoding was assumed to be equal for all age groups and both genders, and thus, the quality of the results remains unaffected. The prevalence of specific inner ear disorders “such as Ménière’s disease, vestibular neuritis, or benign paroxysmal positional vertigo” cannot be estimated from our study findings; however, we presented their specific admission rates, which could be a reflection of the actual prevalence of the diseases. The data of the two medical databases include readmission cases, which could lead to an overestimation of our calculated admission rates. The publicly available data are at the population level (aggregated data) and not at the individual patient level, which restricted our ability to examine the role of important confounding variables on the estimated admission rates.

## 5. Conclusions

In England and Wales, over the past two decades, admissions for inner ear diseases have significantly grown. The likelihood of being admitted for inner ear diseases was higher in females and the middle-aged group. It is necessary to conduct additional cohort studies to find other preventable risk factors and create efficient prevention plans.

## Figures and Tables

**Table 1:** Percentage of admissions from the total number.

ICD Code *	Description	Percentage from Total Number
H80	“Otosclerosis (Otosclerosis involving oval window, nonobliterative, otosclerosis involving oval window, obliterative, cochlear otosclerosis, and unspecified otosclerosis)”	8.8%
H81	“Disorders of vestibular function (Ménière’s disease, benign paroxysmal vertigo, vestibular neuronitis, other peripheral vertigo, vertigo of central origin, and unspecified disorder of vestibular function)”	47.6%
H83	“Other diseases of inner ear (Labyrinthitis, labyrinthine fistula, labyrinthine dysfunction, noise effects on inner ear, and unspecified disease of inner ear)”	43.6%

ICD “International Statistical Classification of Diseases system”; * During the study duration, for vertiginous syndromes in diseases classified elsewhere (H82), there were only six hospital admission episodes in England and no hospital admission episodes in Wales, so H82 data were not considered.

**Table 2:** Percentage change in admission rates for the main inner ear diseases.

Diseases	Admission Rate in 1999 “per 100,000 Persons (95% CI)”	Admission Rate in 2020 “per 100,000 Persons (95% CI)”	Percentage Change
“Disorders of vestibular function”	3.77 (3.60–3.93)	21.00 (20.63–21.37)	457.5%
“Other diseases of inner ear”	5.71 (5.50–5.91)	9.50 (9.25–9.75)	66.5%
“Otosclerosis”	1.47 (1.36–1.57)	1.48 (1.38–1.57)	0.7%

**Table 3:** Percentage change in admission rates for the specific inner ear diseases.

Diseases	Rate of Diseases in 1999 per 100,000 Persons (95% CI)	Rate of Diseases in 2020 per 100,000 Persons (95% CI)	Percentage from Total Number	Percentage Change
“H81.1 Benign paroxysmal vertigo”	0.58 (0.51–0.64)	13.11 (12.82–13.40)	23.2%	2160.3%
“H81.3 Other peripheral vertigo”	0.19 (0.15–0.23)	1.08 (1–1.17)	1.8%	468.4%
“H81.8 Other disorders of vestibular function”	0.07 (0.05–0.1)	0.37 (0.32–0.42)	1.0%	428.6%
“H81.2 Vestibular neuronitis”	0.69 (0.62–0.76)	3.12 (2.98–3.26)	6.8%	352.2%
“H81.9 Unspecified disorder of vestibular function”	0.22 (0.18–0.26)	0.82 (0.74–0.89)	2.2%	272.7%
“H83.8 Other specified diseases of inner ear”	0.1 (0.08–0.13)	0.25 (0.21–0.29)	0.9%	150.0%
“H83.0 Labyrinthitis”	5.27 (5.07–5.47)	9.02 (8.78–9.26)	41.4%	71.2%
“H80.0 Otosclerosis involving oval window, nonobliterative”	0.05 (0.03–0.07)	0.07 (0.05–0.10)	0.3%	40.0%
“H83.1 Labyrinthine fistula”	0.02 (0.01–0.04)	0.03 (0.01–0.04)	0.1%	50.0%
“H80.8 Other otosclerosis”	0.04 (0.02–0.06)	0.06 (0.04–0.08)	0.2%	50.0%
“H81.0 Ménière disease”	1.91 (1.79–2.03)	2.37 (2.25–2.50)	12.0%	24.1%
“H81.4 Vertigo of central origin”	0.11 (0.08–0.14)	0.13 (0.10–0.16)	0.6%	18.2%
“H83.3 Noise effects on inner ear”	0.01 (0–0.01)	0.01 (0–0.02)	0.1%	0.0%
“H83.2 Labyrinthine dysfunction”	0.08 (0.05–0.10)	0.08 (0.05–0.10)	0.4%	0.0%
“H80.1 Otosclerosis involving oval window, obliterative”	0.02 (0.01–0.03)	0.02 (0.01–0.03)	0.1%	0.0%
“H80.9 Unspecified otosclerosis”	1.32 (1.22–1.42)	1.3 (1.21–1.39)	8.0%	−1.5%
“H80.2 Cochlear otosclerosis”	0.03 (0.02–0.05)	0.02 (0.01–0.03)	0.1%	−33.3%
“H83.9 Unspecified disease of inner ear”	0.23 (0.19–0.27)	0.11 (0.09–0.14)	0.7%	−52.2%
“H82.X Unspecified vertiginous syndromes in diseases”	(–)	0 (0–0.01)	0.0%	-

**Table 4:** Change in the admission rate across age groups.

Age Group	Admission Rate in 1999 “per 100,000 Persons (95% CI)”	Admission Rate in 2020 “per 100,000 Persons (95% CI)”	Percentage Change	Percentage from Total Number
Below 15 years	1.19 (0.98–1.41)	1.72 (1.47–1.97)	44.6%	1.42%
15–59 years	9.30 (8.96–9.63)	19.93 (19.46–20.40)	114.3%	42.31%
60–74 years	20.24 (19.18–21.30)	59.38 (57.82–60.94)	193.4%	27.42%
75 years and over	32.29 (30.51–34.07)	124.28 (121.24–127.31)	284.0%	28.84%

## Data Availability

Publicly available datasets were analyzed in this study. This data can be found here: https://digital.nhs.uk/data-and-information/data-tools-and-services/data-services/hospital-episode-statistics, https://www.gov.wales/hospital-admissions-data-online-april-2020-march-2021 (accessed on 14 January 2023).
